# 

**Published:** 1879-05

**Authors:** 


### BOOKS AND PAMPHLETS RECEIVED.

Epitome of Skin Diseases, with Formulæ for Students and Practitioners. By Tilbury Fox, M.D., F.R.C.P., and T. C. Fox, M.B., B.A. Sec. Amer. Ed. Cl., pp. 216. Phil.: H. C. Lea; Chic.: Jansen, McClurg & Co.

Lectures on Practical Surgery. By H. H. Toland, M.D. Sec. Ed. Illustrated. Cl., pp. 520. 1879. Phil.: Lindsay & Blakiston. Chic.: Jansen, McClurg & Co.

The National Dispensatory. By Alfred Stillé, M.D., etc., and J. W. Maisch, PH. D. Leather, pp. 1628, 1879. Phil.: H. C. Lea; Chic.: Jansen, McClurg & Co.

A Clinical Treatise on the Diseases of the Liver. By Dr. F. Theo. Frerichs. In 3 vols. Vol. I. Translated by Ch. Murchison, M.D. Cl., pp. 224. 1879. New York: W. Wood & Co.; Chicago: W. T. Keener.

The Diseases of Live Stock and Their Most Efficient Remedies. By Lloyd V. Tellor. M.D. Cl., pp. 469. 1879. Phil.: D. G. Brinton.

Physiological Therapeutics—A New Theory. By Thos. W. Poole, M.D., M.P.C.S., Ont. Cl. Toronto: Toronto News Co.

A Manual of the Examination of the Eyes. By Dr. E. Landolt. Translated by Swan M. Burnett, M.D. Cl., pp. 307. 1879. Phil.: D. G. Brinton.

The Principles and Practice of Gynæcology. By Thos. Addis Emmet, M.D., etc.; 100 illustrations. Cp., pp. 855. 1879. Phil.: H. C. Lea.

Pathological Report of Montreal General Hospital, 1876–7. Montreal: Dawson Bros.

A Treatise on the Diseases of Infancy and Childhood. By J. Lewis Smith, M.D., etc. Fourth Ed., thoroughly revised. Cp. 8vo, pp. 758. 1879. Phil.: H. C. Lea.

A Guide to the Qualitative and Quantitative Analysis of Urine, designed for Physicians, Chemists and Pharmacists. By Dr. G. Neubauer. (Translation.) Revised by Ed. S. Wood, M.D. Leather, pp. 551. New York: W. Wood & Co.

Report of the U. S. Marine Hospital Service, 1876–7. Cl., pp. 213. 1878.

Address delivered before the American Academy of Dental Sciences at their Eleventh Annual Meeting held at Boston, Oct. 30, 1878. By Chas. M. Eliot, LL.D.

Oyster Shuckers' Corneitis. Reprint from *Virginia Medical Monthly*, Feb., 1879. By W. J. McDowell, M.D.

Influences of Altitudes on Consumptives. By J. Hilgard Tyndale, M.D. Reprint from *St. Louis Courier of Medicine.*

Valedictory Address to the Graduating Class of Jefferson Medical College at the Fifty-fourth Annual Commencement, delivered in the Academy of Music, March 12, 1879. By J. A. Meiggs.

The Causes of Sudden Death of Puerperal Women. An Address in Obstetrics and Diseases of Women and Children, delivered before the American Med. Ass'n, June 5, 1878.

Opium as a Tonic and Alterative and its Hypodermic Use in the Debility and Amaurosis Sometimes Consequent upon Onanism. By B. A. Pope, M.D.

The Treatment of Dropsy of the Gall Bladder by Operation (Cholocystotomy), with Notes of a Successful Case. By G. Brown, M.R.C.S., L.S.A. Reprint from *British Med. Journal.*

A Letter to Thomas P. Rogers, of the Twenty-eighth Representative District, in the Thirty-first General Assembly of the State of Illinois. By Andrew McFarlane, M.D.

Constitution and List of Members of the American Public Health Association.

Maternal Impressions; Mothers' Marks. An Exposé of a Popular Fallacy. By Roswell A. Park, A.M., M.D. Rep. from *Southern Clinic*, Feb.. 1879.

The Etiology and Pathology of Chronic Joint Disease. By Newton M. Shaffer, M.D. (Series of American Clinical Lectures. Edited by E. C. Seguin.

### ANNOUNCEMENTS FOR THE MONTH.

### SOCIETY MEETINGS.

Chicago Medical Society—Mondays, May 5 and 19.

West Chicago Medical Society—Mondays, May 12 and 26.

### CLINICS.

MONDAY.

Eye and Ear Infirmary—2 p. m., Ophthalmological, by Prof. Holmes; 3 p. m., Otological, by Prof. Jones.

Mercy Hospital—1:30 p. m., Surgical, by Prof. Andrews.

Rush Medical College—2 p. m., Dermatological and Venereal, by Prof. Hyde; 3 p. m., Medical, by Dr. Bridge.

Woman's Medical College—2 p. m., Dermatological, by Dr. Maynard.

TUESDAY.

Cook County Hospital—2 to 4 p. m., Medical and Surgical Clinics.

Mercy Hospital—1:30 p. m., Medical, by Prof. Hollister.

WEDNESDAY.

Chicago Medical College—1:30 p. m., Eye and Ear, by Prof. Jones.

Rush Medical College—3:30 to 4:30 p. m., Diseases of the Chest, by Dr. E. Fletcher Ingals.

THURSDAY.

Chicago Medical College—1:30 p. m., Medical, by Prof. Quine.

Rush Medical College—3 p. m., Diseases of the Nervous System, by Prof. Lyman.

Eye and Ear Infirmary—2 p. m., Ophthalmological, by Dr. Hotz.

FRIDAY.

Cook County Hospital—2 to 4 p. m., Medical and Surgical Clinics.

Mercy Hospital—1:30 p. m., Medical, by Prof. Davis.

SATURDAY.

Rush Medical College—2 p. m., Surgical, by Prof. Gunn.

Chicago Medical College—2 p. m., Surgical, by Prof. Isham; 3 p. m., Neurological, by Prof. Jewell.

Woman's Medical College—11 a. m., Ophthalmological, by Dr. Montgomery.

Daily Clinics, from 2 to 4 p. m., at the Central Free Dispensary, and at the South Side Dispensary.

